# Book Reviews

**Published:** 1897-12

**Authors:** 


### Book Reviews.

INTERNATIONAL CLINICS. Vol. II., Seventh Series. J. B. Lippincott & Co., Phila., 1897.

This volume of International Clinics contains contributions from the following: C. S. Bacon, J. W. Baldy, S. Baruch, L. L. Bishop, C. H. Burnett, W. E. Casselberry, Jno. G. Cecil, E. Treacher Collins, C. G. Cumston, J. M. DaCosta, Sir Dyce Duckworth, W. S. Tenwick, L. Webster Fox, A. Pearce Gould, H. H. Grant, G. E. Herman, Jas. B. Herrick, J. O. Hirschfelder, W. C. Hollopeter, Edward Jackson, Alex. James, A. O. J. Kelly, J. H. Lloyd, G. H. McKenzie, G. H. Makuen, H. D. Marcus, E. E. Maylard, Thos. J. Mays, Henry Morris, Ohmann Dumernil, Thos. Oliver, S. D. Risley, Jno. B. Roberts, W. L. Rodman, J. L. Stenen, J. M. Taylor, Norman Walker, W. H. Wathen, Jas. Whittaker, and C. A. Wood.

Their contributions cover the various provinces of medicine, including treatment, medicine, neurology, surgery, gynæcology, and obstetrics; ophthalmology, laryngology, pharyngology, rhinology, otology, and dematology. The volume as usual is well illustrated.

TRAUMATIC INJURIES OF THE BRAIN AND ITS MEMBRANES. With a special study of Pistol-shot Wounds of the Head in their medico-legal and surgical relations. By Charles Phelps, M. D., Surgeon to Bellevue and St. Vincent's Hospitals. With forty-nine illustrations. New York. D. Appleton & Company, 1897.

This work, which is the result of painstaking and accurate observation extending over a long period of time, promises to become an authority on this class of injury, especially in its medico-legal relation. The nature of wounds, of entrance and exit, condition of soft parts, presence or absence of burns, of powder stains, of grains of powder free and imbedded, are the questions principally concerned in the book.

Each condition is illustrated by a full page photograph taken from nature and showing the effect of pistol-shot injuries at several ranges as well as in contact, and with revolvers of different calibre.

These observations are necessarily of value as throwing light on cases involving the question of homicidal or self-inflicted injury. The book will be welcomed by surgeons and general practitioners as well, as the latter are not infrequently called upon to testify in court upon precisely such cases as are illustrated and treated of by Dr. Phelps.

In addition to the treatise proper, there is given a brief history of three hundred cases of intracranial traumatism, two hundred and twenty-five of which were verified by autopsy.

ESSENTIALS OF OBSTETRICS. By Charles Jewett, A. M., M. D. ScD., Professor of Obstetrics and Pediatrics in the Long Island College Hospital, and Obstetrician to the Hospital. Assisted by Harold F. Jewett, M. D. Illustrated by eighty wood cuts and three colored plates. Lea Brothers & Company. New York and Philadelphia, 1897.

This book, as is indicated by the title, is designed to present the essential principles of obstetrics in a form sufficiently condensed as to be readily available to the student of medicine, and to the practitioner as well.

Works of this character save a great deal of time to one who lacks inclination or opportunity to wade through more elaborate treatises in order to get the information he particularly desires, and they are especially to be commended to the busy man.

This work is practical and keeps that purpose constantly in view, so that there is but little which is theoretical in its 350 pages. Altogether, it will be found helpful and instructive.

---

TWENTIETH CENTURY OF PRACTICE. Mental Diseases—Diseases of Childhood and old age. William Wood & Co.

The opening article on Insanity contributed by G. Fielding Blanford is far from complete; the various types receive but a cursory glance and an unfamiliar classification renders even this glance unsatisfactory.

A much more acceptable chapter is that on Idiocy by Paul Lollier, and the fifty pages occupied by Lombroso on Criminal Anthropology represent in a condensed form the study of a lifetime.

The rest of the volume is taken up by an article on Senility by Boy-Tessier, and one on the Diseases of Childhood by Jules Comby; it is for the last of these that the book will be most read.

